# Electrochemical Biochip Assays Based on Anti-idiotypic Antibodies for Rapid and Automated On-Site Detection of Low Molecular Weight Toxins

**DOI:** 10.3389/fchem.2019.00031

**Published:** 2019-02-01

**Authors:** Katharina Schulz, Christopher Pöhlmann, Richard Dietrich, Erwin Märtlbauer, Thomas Elßner

**Affiliations:** ^1^Bruker Daltonik GmbH, Leipzig, Germany; ^2^Department of Veterinary Sciences, Faculty of Veterinary Medicine, Ludwig-Maximilians-Universität München, Munich, Germany

**Keywords:** electrochemical biochip, on-site detection, pBDi, competitive immunoassay, anti-idiotypic antibodies, aflatoxins, T-2 toxin, saxitoxin

## Abstract

Phycotoxins and mycotoxins, such as paralytic shellfish poisoning toxins, type A trichothecenes, and aflatoxins are among the most toxic low molecular weight toxins associated with human poisoning incidents through the consumption of naturally contaminated food. Therefore, there is an utmost need for rapid and sensitive on-site detection systems. Herein, an electrochemical biochip for fast detection of saxitoxin, T-2 toxin as well as aflatoxin M1 and their corresponding congeners, respectively, using a portable and fully automated detection platform (pBDi, portable BioDetector integrated) was developed. Toxin analysis is facilitated upon the biochip via an indirect competitive immunoassay using toxin-specific antibodies combined with anti-idiotypic antibodies. The developed biochips enable detection in the low ng/mL-range within 17 min. Moreover, the assays cover a wide linear working range of 2–3 orders of magnitude above the limit of detection with an inter-chip coefficient of variation lower than 15%. The broad specificity of the employed antibodies which react with a large number of congeners within the respective toxin group allows efficient screening of contaminated samples for the presence of these low molecular weight toxins. With respect to the analysis of human urine samples, we focused here on the detection of saxitoxin, HT-2 toxin, and aflatoxin M1, all known as biomarkers of acute toxin exposure. Overall, it was proved that the developed biochip assays can be used to rapidly and reliably identify severe intoxications caused by these low molecular weight toxins.

## Introduction

Low molecular weight toxins, like phycotoxins and mycotoxins, are highly toxic contaminants posing a risk to human and animal health. Intoxication occurs through ingestion of contaminated food, feed or from environmental exposure. Consumption of contaminated seafood leads to paralytic shellfish poisoning (PSP) caused by PSP toxins (Anderson et al., 2012). PSP toxins are a class of chemically related neurotoxins comprising up to 50 congeners which differ significantly in toxicity, with saxitoxin (STX) being the most toxic one. Symptoms of PSP include numbness of lips, headache, dizziness, nausea, vomiting, and diarrhea followed by muscle paralysis and respiratory failure in acute cases (Van Egmond et al., 1993). Another health threat is associated with the consumption of plant food contaminated by fungi which produce mycotoxins as secondary metabolites. Trichothecenes and aflatoxins are two classes of mycotoxins mostly associated with human health issues. T-2 toxin (T-2) and HT-2 toxin (HT-2) belong to the type A trichothecenes. Moreover, T-2/HT-2 are the most potent trichothecene toxins and poisoning in humans is known as alimentary toxic aleukia resulting in alimentary hemorrhage, damages to hematopoietic tissues, and vomiting (Adhikari et al., 2017). For the group of aflatoxins, five different aflatoxins have been considered to be important for food safety including aflatoxin B1 (AFB1), aflatoxin B2 (AFB2), aflatoxin G1 (AFG1), aflatoxin G2 (AFG2), and the AFB1 metabolite aflatoxin M1 (AFM1). Due to carcinogenetic properties of AFB1, a dietary exposure to AFB1 can be linked to the development of hepatocellular carcinoma, while an acute aflatoxicosis induces abdominal pain, vomiting, edema, and death (Williams et al., 2004).

For diagnosis of an acute poisoning incident, two approaches can be used: (i) analysis of suspected food or (ii) analysis of biomarkers in body fluids. If food is no longer available as sample matrix, the only chance to obtain a hint for an intoxication is to analyze body fluid samples of affected persons for the presence of the parent toxin or metabolites. One of the most common body fluids for biomarker analysis is urine due to the non-invasive sampling. Multiple studies demonstrated that ingested PSP toxins are excreted by urinary routes (Gessner et al., 1997; García et al., 2004). The same applies for T-2 and aflatoxins. T-2 is rapidly metabolized and, in animals, the most typical metabolites of T-2 are HT-2, T-2 triol, T-2 tetraol as well as their hydroxylated variants. All compounds are found in urine and feces of exposed animals (Wu et al., 2010). AFM1 is a main metabolite of AFB1 and can be used as a valid indicator of exposure to aflatoxins due to a dose-dependent relationship between the excretion of AFM1 in urine and the uptake of AFB1 (Groopman and Kensler, 1993).

Detection of these low molecular weight toxins is often achieved by methods utilizing chromatographic and/or mass spectrometry-based approaches (Turner et al., 2009). These methods require skilled personnel, expensive laboratory equipment and complex preparation steps, and, thus, they are time-consuming and laborious. To provide a rapid and sensitive detection based on cost-effective and easy-to-use methods, a variety of screening methods has been developed. These methods mainly rely on antigen-antibody reactions. The most widely used immunoassays include microplate-based immunoassays, immunochromatographic assays (or lateral flow assays, LFA) as well as different immuno-biosensors. LFAs have several advantages over microplate-based assays including low cost as well as rapidity and simple use; on the other hand application of LFAs may be restricted by insufficient analytical sensitivity (Posthuma-Trumpie et al., 2009). The increasing emergence of immuno-biosensors in the field of food analysis is based on their advantageous properties such as being highly sensitive, portable, robust and capable for automation (McGrath et al., 2012). Optical immunosensors use, for example, surface plasmon resonance (SPR) for detection. However, unlabeled assay formats such as SPR suffer often from limited sensitivity and matrix interference (Granqvist et al., 2013). In contrast, labeled assay formats are characterized by improved sensitivity, whereas a disadvantage is the additional labeling step required. McNamee et al. (2014) developed a planar waveguide microarray for multiplex detection of five groups of harmful phycotoxins applying 15 min assay time. Besides optical methods, electrochemical transduction technologies for immuno-biosensors were intensively investigated for analysis of low molecular weight toxins (Vidal et al., 2013). Advantages of electrochemical biosensors include their sensitivity, selectivity, low cost, simplicity, and in particular the potential for miniaturization and portability as well as integration in automated devices (Farré et al., 2009). Portability is an important feature for immuno-biosensors because it allows point-of-care or on-site testing for medical diagnostics, food, and environmental monitoring. Electrochemical immuno-biosensors were established for several low molecular weight toxins in the past, for example for AFB1 (Lin et al., 2015), or STX (Bratakou et al., 2017).

In general, detection of low molecular weight toxins by immunoassay-based techniques relies on a competitive format, in which the toxin is either coupled to carrier proteins used for coating or labeled with enzyme competing with the toxin in the sample for a limited amount of capture antibodies. Unfortunately, the chemical coupling of the toxin to proteins inevitably exposes operators and the environment to the toxic reagents, whereas anti-idiotypic antibodies have a promising potential to replace these hapten-protein conjugates (He et al., 2010). Anti-idiotypic antibodies of the β-type are raised against the paratope (antigen-binding site) of the primary anti-hapten antibody such displaying an “internal image” of the hapten (Jerne, 1974). In the final assay, these anti-idiotypic antibodies compete with the original hapten for binding sites of anti-hapten antibody. In the past, both polyclonal and monoclonal antibody techniques have been used to successfully generate anti-idiotypic antibodies against haptens like mycotoxins (Chanh et al., 1991). Moreover, recombinant antibody approaches have also been applied to generate and genetically engineer anti-idiotypic antibodies (Wang et al., 2013). In competitive immunoassay formats anti-idiotypic antibodies can serve either as capture antigen (Shu et al., 2015) or as competing reagent (Shu et al., 2016). Furthermore, anti-idiotypic antibodies have been utilized as surrogate toxin for the establishment of a standard curve (Guan et al., 2011). With regard to the development of immuno-biosensors, Szkola et al. (2014) demonstrated the successful implementation of anti-idiotypic antibodies as capture molecules on a chemiluminescence-based microarray for detection of STX.

In the present study, electrochemical readable anti-idiotypic antibody based competitive immunoassays allowing the detection of the phycotoxin STX and the mycotoxins T-2 as well as AFM1 and corresponding congeners, respectively, using a portable and fully automated detection platform (pBDi, portable BioDetector integrated) (Pöhlmann and Elßner, 2018) were developed. This electrochemical detection platform with corresponding biochips based on a non-competitive immunoassay format has already been tested for detection of high molecular weight toxins such as ricin (Worbs et al., 2015) and staphylococcal enterotoxin B (Nia et al., 2016) in the field of biosecurity. Here, we established competitive biochip-based assays as a screening tool to identify STX, HT-2 and AFM1 as urinary biomarker for diagnosing an acute poisoning incident, for example, after consumption of contaminated food.

## Materials and Methods

### Materials

#### Reagents

Phosphate-buffered saline (PBS; 10 mM disodium hydrogen phosphate, 1.8 mM potassium dihydrogen chloride, 137 mM sodium chloride, 2.7 mM potassium chloride) pH 7.4, Tween-20, trehalose, sorbitol, magnesium chloride hexahydrate (MgCl_2_), skimmed milk powder, sodium hydroxide (NaOH), sodium dodecyl sulfate (SDS), *p*-aminophenyl-β-D-galactopyranoside (*p*-APG), streptavidin-β-D-galactosidase, and bovine serum albumin (BSA) were purchased from Sigma-Aldrich GmbH (Taufkirchen, Germany). Ultrapure water was obtained from a Milli-Q purification system (Millipore, Billerica, MA, USA).

#### Toxin Standards

The PSP toxin standards, including STX, decarbamoylsaxitoxin (dc-STX), decarbamoylneosaxitoxin (dc-NEO), gonyautoxins 1 and 4 (GTX-1/-4), decarbamoylgonyautoxins 2 and 3 (dc-GTX-2/-3) and N-sulfocarbamoyl-gonyautoxins 2 and 3 (C1/C2) were purchased from LabMix24 GmbH (Hamminkeln, Germany), whereas neosaxitoxin (NEO), gonyautoxins 2 and 3 (GTX-2/-3) as well as gonyautoxin 5 (GTX-5) were from CIFGA S.A. (Lugo, Spain). The T-2 standards, including T-2 and its metabolites HT-2, T-2 triol, and T-2 tetraol were obtained from Romer Labs (Getzersdorf, Austria). The aflatoxin standards AFM1, AFB1, AFG1, AFB2, and AFG2 were purchased from Sigma-Aldrich GmbH (Taufkirchen, Germany).

### Methods

#### Antibody Production

Monoclonal antibodies (mAb), specific for low molecular weight toxins, 5F7 (anti-STX), 2A12 (anti-T-2/HT-2), and 2D1 (anti-AFM1) were prepared as described (Dietrich et al., 1995). The β-type anti-idiotypic mAbs 1D8 (anti-5F7), 1D6 (anti-2A12), and 1G10 (anti-2D1) were generated according to Szkola et al. (2014).

#### Biotinylation of Detection Antibodies

Detection mAbs were biotinylated using EZ-Link™ Sulfo-NHS-LC-Biotin (Thermo Fischer Scientific, Waltham, MA, USA) dissolved in ultrapure water. The antibodies were mixed with a 20-fold molar excess of the biotin reagent and incubated for 1 h at room temperature. Excess of non-reacted biotin reagent was removed using Zeba™ Spin 7K MWCO Desalting Columns (Thermo Fisher Scientific, Waltham, MA, USA) equilibrated with PBS. The concentration of biotinylated antibody was determined based on absorbance measurements at λ = 280 nm using the NanoPhotometer NP-80 (Implen GmbH, Munich, Germany).

#### Immobilization of Antibodies on Gold Electrodes of Biochips

The biochips were manufactured on 8-inch silicon wafers at the Fraunhofer Institute for Silicon Technology (Itzehoe, Germany) as described (Elsholz et al., 2006). The final biochip size was 9 × 10 mm carrying 16 interdigitated array (IDA) gold electrodes each with a diameter of 500 μm. Capture mAbs were immobilized directly via physisorption onto the gold electrodes using a non-contact piezo-electronic spotter (sciFLEXARRAYER S3, Scienion Inc., Berlin, Germany) at a final concentration of 400 μg/mL 1D8, 100 μg/mL 2A12, and 400 μg/mL 2D1 in 0.4% (w/v) BSA/PBS based on the results of preceding optimization experiments. BSA was added as co-immobilization agent for stabilization of capture mAbs immobilized on gold electrode surface. Biotinylated rabbit anti-mouse IgG (Sigma-Aldrich GmbH, Taufkirchen, Germany) was used as positive control (2.5 μg/mL in 0.02% (w/v) BSA/PBS) and 400 μg/mL BSA/PBS as negative control. 130 droplets were spotted on each position resulting in a total volume of ca. 50 nL per electrode. After spotting, the biochips were incubated in a humidity chamber with PBS for 2 h at room temperature, followed by a 16 h-incubation at 4°C. Afterwards, the biochips were washed with 0.025% (v/v) Tween-20/PBS and PBS and then, blocked for 30 min with 1% (w/v) skimmed milk powder/PBS to prevent unspecific binding events. Following a final wash step with ultrapure water, spotted biochips were evaporated to dryness and stored at 4°C protected from light. Finally, the biochip was mounted in a polycarbonate cartridge consisting of an internal flow cell with a volume of 10 μL.

#### Instrumentation and Biochip Measurement

Spotted biochips were measured using the fully automated, electrochemical detection platform pBDi (Bruker Daltonik GmbH, Leipzig, Germany). The electrochemical current measurement is calculated by applying a potential of +150 mV to the anodic fingers and −400 mV to the cathodic fingers of the IDA gold electrodes. The multipotentiostat achieves a resolution of the current of 5 pA within a range of ±200 nA.

As sample, a total volume of 800 μL toxin standard sample or urine sample mixed with the respective detection antibody was applied. The automatic assay program is summarized in Table 1 indicating a total assay time of 16.7 min. It includes, amongst other steps, washing with assay buffer (PBS pH 7.4, 1% (w/v) BSA, 0.025% (v/v) Tween-20), a competition step of the toxin sample with biotinylated detection antibody for limited binding sites of the capture antibody, an incubation step with reporter enzyme (streptavidin-β-D-galactosidase stabilized with 100 mM MgCl_2_ and 100 mM sorbitol, then diluted to 1 U/mL with assay buffer) to label bound detection antibody, and a final signal generating step applying the reporter enzyme substrate *p*-APG (diluted to 1 mg/mL with assay buffer). Monitoring of the generated current by enzymatic substrate conversion as well as redox cycling was performed according to Elsholz et al. (2006) in stopped-flow mode. The absolute current slope for each electrode position is determined by linear regression during the first 4 s with 1 s delay after stopped-flow. For better inter-chip reproducibility, the absolute current slopes of target electrode positions were mathematically normalized to the signal of the spotted positive control (Pos.Co.) and negative control (Neg.Co.):

$$\begin{array}{l} \text{Normalized}\;\text{signal}\;\left(\text{\%}\right)=100\times \left[\frac{\text{Slope}\left(\text{Target}\right)-\text{Slope}(\text{Neg}.\text{Co}.)}{\text{Slope}\left(\text{Pos}.\text{Co}.\right)-\text{Slope}(\text{Neg}.\text{Co}.\;)}\right] \end{array}$$

Normalized signals were used for further data analysis.

**Table 1:** Automated assay program for the detection of STX, T-2/HT-2, and AFM1.

**Step**	**Process**	**Duration (s)**	**Temperature (**°**C)**
1	Equilibration with assay buffer	56	32
2	Sample and detection antibody flow	20	42
3	Competition reaction	594	42
4	Wash flow with assay buffer	26	42
5	Reporter enzyme flow	20	42
6	Reporter enzyme incubation	180	42
7	Wash flow with assay buffer	40	42
8	Substrate flow	10	50
9	Stopped flow	15	50
10	Wash flow with assay buffer	41	32

To prevent carry-over, a 4 min-wash step with 0.1% (w/v) SDS in 0.1 M NaOH was performed before next measurements.

#### Preparation of Dose-Response Curves

Stock solutions of STX, T-2 and HT-2 as well as AFM1 were used to prepare different working solutions in assay buffer ranging from 0 to 300 ng/mL for STX and AFM1, 0–400 ng/mL for T-2 and 0–1,000 ng/mL for HT-2. Working solutions were then mixed with the respective detection antibody (STX: 50 ng/mL 5F7-bio, T-2/HT-2: 150 ng/mL 1D6-bio, AFM1: 300 ng/mL 1G10-bio). According to the automated assay program, a 1 min pre-incubation of the toxin and the detection antibody takes place at room temperature before pumping to the biochip.

#### Assay Specificity

Specificity was evaluated by incubating the biochip with the respective toxin group congeners in presence of the corresponding detection antibody (STX: 50 ng/mL 5F7-bio, T-2/HT-2: 150 ng/mL 1D6-bio, AFM1: 300 ng/mL 1G10-bio). For PSP toxin detection, 150 ng/mL STX, dc-STX, NEO, dc-NEO, GTX-1/-4, GTX-2/-3, dc-GTX-2/-3, C1/C2 and GTX-5, respectively, were applied. For the T-2/HT-2 biochip, 150 ng/mL T-2 and equal concentrations of its metabolites HT-2, T-2 triol and T-2 tetraol were used. For aflatoxin detection, 10 ng/mL AFM1, AFB1, AFG1, AFB2, and AFG2, respectively, were incubated on the biochip. All toxin samples were diluted in assay buffer for measurements.

#### Data Analysis

Dose-response curves were described by the 4-parameter logistic linear regression model using GraphPad Prism 6 (GraphPad Software, La Jolla, CA, USA). Displayed error bars indicate the standard deviation and were obtained from different biochips as indicated. The limit of detection (LOD), linear working range, and midpoint value (IC_50_) were interpolated from the 4-parameter logistic function. The LOD corresponds to the toxin concentration which gave a normalized signal equal to the normalized signal when no toxin is present (B0) minus three times its standard deviation obtained from six biochips with two target electrode positions. The linear working range was determined as the concentrations of the toxin reducing the B0 signal to 20–80%. The IC_50_ were calculated as the toxin concentration that lowered the normalized signal to 50% of B0. For verification of assay specificity, percent inhibition was calculated by the formula:

$$\begin{array}{l} \text{Inhibition}\;(\text{\%})=100-\;\left[\frac{\text{Signal}\;\times 100}{\text{B}0\;\text{Signal}}\right] \end{array}$$

#### Preparation of Urine Samples

First morning urine was used and collected freshly each day from healthy volunteers. One mL urine was mixed with 40 ng/mL STX or 20 ng/mL HT-2 or 8 ng/mL AFM1 and incubated for 30 min at room temperature using an end-over-end mixer. Then, urine samples as well as blank urine samples were centrifuged at 4,500 × g for 5 min, diluted 4-fold with assay buffer, mixed with the respective biotinylated detection antibody and applied to the biochip.

#### Threshold Value T and Cut-Off Factor Fm for Urine Samples

To demonstrate the applicability of the assays as screening methods for the analysis of urine samples, threshold value T and cut-off value Fm were determined for each toxin with an error probability of 5% according to the Decision No 2002/657/EC (EC, 2002; CRLs, 2010). Therefore, 20 urine samples spiked with toxin and 20 blank urine samples were analyzed. The obtained signals of the blank and spiked urine samples were set in relation to the B0 signal observed in buffer. The threshold value T was calculated as the mean signal of blank samples minus 1.729 times its standard deviation. The cut-off factor Fm was estimated as being the mean signal of spiked urine samples plus 1.729 times the standard deviation. A competitive method is validated as a screening method, when Fm < T.

## Results

### Assay Principle

**Figure 1:**
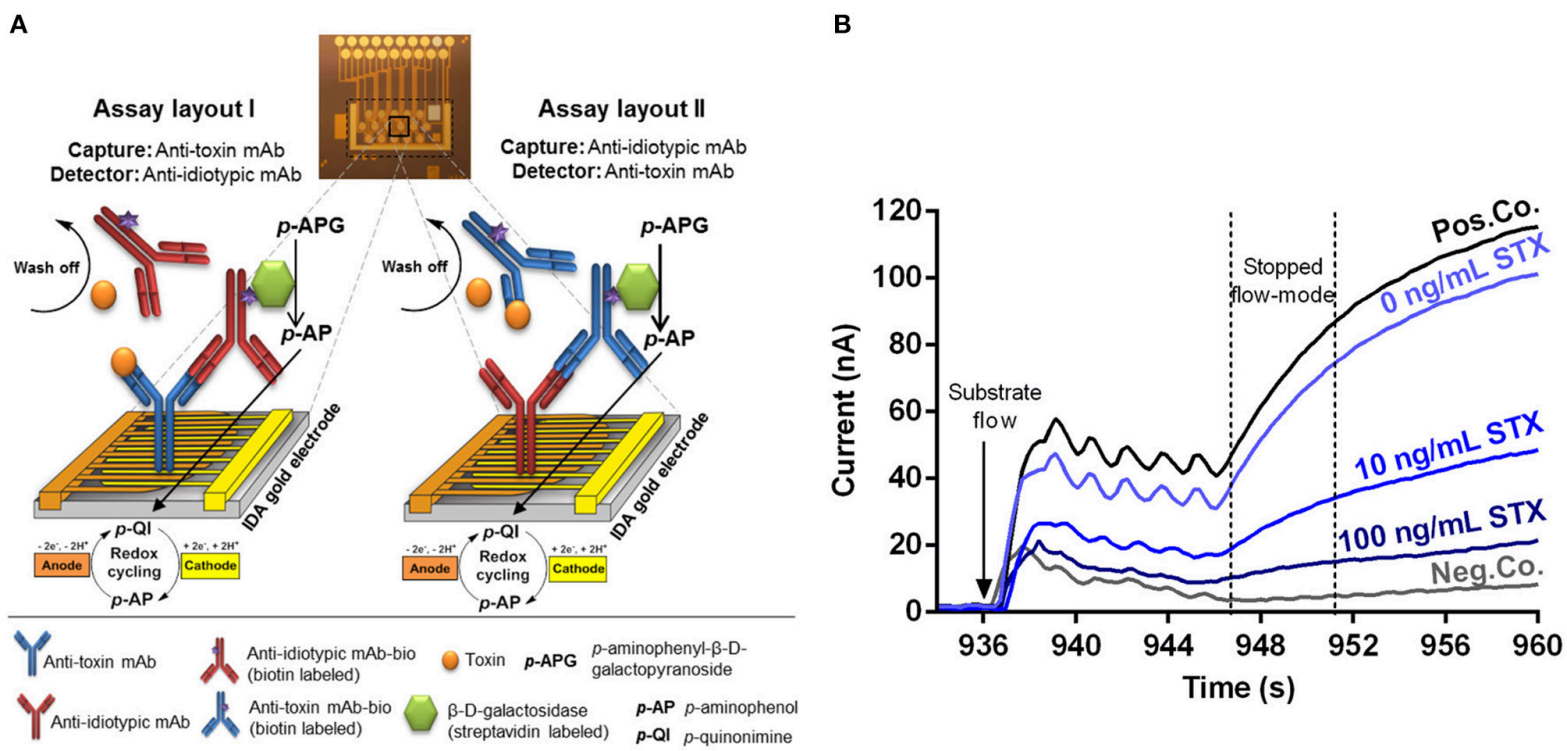
Illustrated principle of the competitive, anti-idiotypic antibody-based biochip assays with amperometric measurement. **(A)** The electrical biochip exhibits 16 IDA gold electrodes for immobilization of capture molecules. Utilization of anti-toxin mAbs and anti-idiotypic mAbs as capture or detector enabling two different assay layouts (I and II) based on a competitive format. During an automatic measurement the sample (toxin) plus the biotinylated detector antibody, the streptavidin labeled β-D-galactosidase and the substrate *p*-APG are sequentially applied to the biochip. Signal amplification of electrochemical detection is realized via redox cycling of *p*-AP between anodic and cathodic fingers of the electrode. **(B)** Amperometric response curves (current vs. time) were generated after substrate flow to the biochip followed by enzymatic conversion to the electrochemically active product and redox cycling in stopped-flow mode. Curves for 10 and 100 ng/ml STX were obtained and compiled from two independent biochips. Pos.Co. = Biotin labeled rabbit anti-mouse IgG; Neg.Co. = BSA.

A competitive electrochemical immunoassay format relying on the use of anti-idiotypic mAbs for detection of several low molecular weight toxins such as STX, T-2/HT-2, and AFM1 is presented. In contrast to traditional competitive immunoassays, the use of anti-idiotypic mAbs circumvents the need for toxin-protein conjugates either for coating or as tracer molecules. Either anti-toxin mAbs (layout I) or anti-idiotypic mAbs (layout II) are immobilized onto the IDA gold electrodes by physisorption (Figure 1A). In layout I, samples containing the toxin and biotinylated anti-idiotypic mAbs are applied simultaneously to the coated biochip. Free toxin in the sample and biotinylated anti-idiotypic mAbs compete for limited binding sites of the immobilized anti-toxin mAbs. The biotinylated anti-idiotypic mAb serves as detector replacing the toxin-enzyme conjugate needed for competition in traditional competitive immunoassay format. In layout II, the anti-idiotypic mAb is immobilized onto the gold electrodes, thus, no toxin-protein conjugate is required for immobilization allowing safe manufacturing of biochips. Following the application of the toxin sample and the biotinylated anti-toxin mAb, a competition between the free toxin and the immobilized anti-idiotypic mAb for binding to the biotinylated anti-toxin mAbs functioning as detector takes place. The higher the toxin concentration, the fewer detection antibodies can bind to immobilized capture antibodies. Detection is realized via biotin-streptavidin interaction of biotinylated detection antibodies with the streptavidin labeled reporter enzyme β-D-galactosidase. Then, enzymatic conversion of the electrochemically inactive substrate *p*-APG to the electrochemically active product *p*-aminophenol (*p*-AP) occurs. Moreover, the arrangement of the electrodes as interdigitating anodic and cathodic fingers enables a redox cycling after substrate conversion. The electrochemically active product *p*-AP is oxidized at the anodic fingers and the oxidation product, *p*-quinonimine (*p*-QI), is then reduced back to *p*-AP at the cathodic fingers resulting in a signal amplification factor of approximately 10 (Nebling et al., 2004). Generally, for both assay layouts, the generated current signal is inversely proportional to the toxin concentration of the sample due to the underlying applied competitive assay principle (Figure 1B). Pos.Co. position shows a sharp increase of the current vs. time during stopped-flow mode, whereas the Neg.Co. position exhibits a constant low current signal.

### Selection of Capture and Detection Antibodies

The following parameters were optimized independently for the detection of the three low molecular weight toxins: (i) assay layout (layout I vs. layout II, see Figure 1A), (ii) concentration of immobilized capture mAb, and (iii) concentration of biotinylated detection mAb to achieve most sensitive assays.

**Figure 2:**
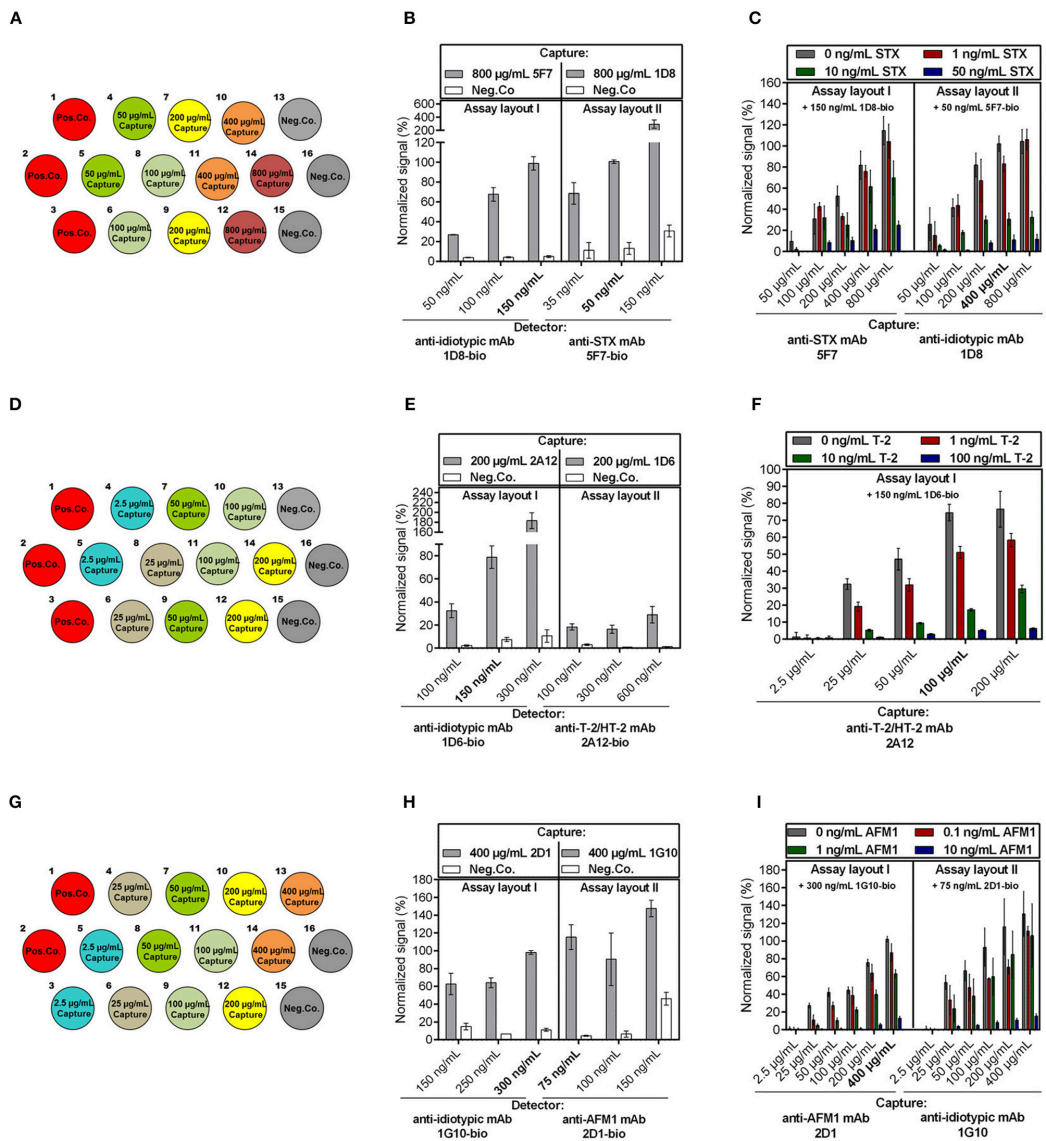
Optimization of the anti-idiotypic antibody-based biochip assay focusing on capture and detection antibody concentration. Anti-toxin mAbs as well as anti-idiotypic mAbs were spotted as capture antibodies with varying concentrations as indicated for **(A)** STX, **(D)** T-2/HT-2, and **(G)** AFM1. **(B,E,H)** Influence of the detection antibody concentration in absence of toxin on the signal response for each assay setup is exemplarily shown for electrode positions spotted with the highest capture concentration and Neg.Co. for the **(B)** STX, **(E)** T-2/HT-2, **(H)** AFM1 assay. Chosen concentrations of detection mAb are indicated bold. **(C,F,I)** Influence of the capture antibody concentration on the signal response for each assay layout applying a fixed concentration of detection mAb in the presence and absence of different concentrations of **(C)** STX, **(F)** T-2, and **(I)** AFM1. Toxin concentrations and applied detection mAb concentration are stated in the figures. Chosen capture mAb concentrations of the most sensitive assay layout are highlighted. Error bars represent standard deviation from two biochips with two target electrode positions (*n* = 4).

First, anti-toxin mAbs and anti-idiotypic mAbs were tested for their suitability to be used as capture or detector on the biochip. Therefore, the anti-toxin mAbs and anti-idiotypic mAbs were spotted in duplicate on three different biochips, in different concentrations ranging from 2.5 μg/mL up to 800 μg/mL (Figures 2A,D,G). For optimization of detector concentration, each biochip was exposed to different concentrations of the respective detection antibody (in the range from 35 to 600 ng/mL) in the absence of toxin. To demonstrate the influence of different detector concentrations on the signal response, results are exemplarily shown for electrode positions spotted with the highest capture concentration and with Neg.Co. (Figures 2B,E,H). In most cases, signal intensity increased with the concentration of detection antibody applied (Figures 2B,E left,**H**). For STX assay layout II, signal intensity increased with rising concentrations of biotinylated detector antibody (35, 50, 150 ng/mL), however, use of biotinylated anti-STX mAb 5F7-bio resulted in elevated signals on Neg.Co. electrode positions covered with BSA (Figure 2B right). Similar results were obtained for the AFM1 assay layout II in which application of 150 ng/mL anti-AFM1 mAb 2D1-bio also led to a signal rise of the Neg.Co. electrode positions, whereas use of 75 ng/mL or 100 ng/mL 2D1-bio, respectively, did not result in an elevation of negative control signals (Figure 2H right). Contrarily, in the T-2 assay this effect was not observed. However, further experiments revealed that activity of 2A12-bio in assay layout II was clearly impaired (data not shown) indicating that mAb 2A12 is not suitable for biotinylation in the described manner and thus, is not applicable as detector. Therefore, T-2 assay layout II was not further evaluated. Optimal detection antibody concentration was chosen based on the selection criterions (i) a maximum signal response in combination with (ii) a low background signal. Therefore, detection antibody concentrations were selected as follows: 150 ng/mL 1D8-bio (STX assay layout I), 50 ng/mL 5F7-bio (STX assay layout II), 150 ng/mL 1D6-bio (T-2 assay layout I), 300 ng/mL 1G10-bio (AFM1 assay layout I), and 75 ng/mL 2D1-bio (AFM1 assay layout II).

Second, different dilutions of the corresponding toxin in combination with optimal detector concentration were applied to the biochips to select the optimal capture concentration (Figures 2C,F,I). In all cases, signal intensity correlated with the amount of capture antibody, which indicates that the higher the concentration of capture molecules, the higher the overall signal intensity. Furthermore, due to the competitive nature of the electrochemical immunoassay, decreasing signal intensities were obtained at increasing toxin concentrations. Overall, the results, summarized in Figure 2, showed that the sensitivity of the particular assays depends on the applied concentration of the capture antibody. For instance, after immobilizing 800 μg/mL anti-idiotypic mAb 1D8 (STX assay layout II), a clear differentiation of 0 and 1 ng/mL STX was not possible (Figure 2C right) while it was possible at lower coating concentrations. A possible explanation is that at higher coating concentrations the gold electrode is highly covered with anti-idiotypic antibodies, resulting in too many free binding sites for the selected optimal detection mAb concentration causing a loss in sensitivity. Analogously, immobilization of 100 μg/mL anti-T2/HT-2 mAb 2A12 resulted in a more significant differentiation of 0 and 1 ng/mL T-2 compared to immobilization of 200 μg/mL 2A12 (Figure 2F).

Third, for selection of optimal assay layout, the chosen selection criterions were (i) the largest difference between the signals obtained in the absence and presence of the toxin and (ii) a relative standard deviation of the signal response between the two biochips ≤ 20% for the indicated concentration. Particularly, the AFM1 assay layout II exhibited signals with significantly higher standard deviations than signals in assay layout I (Figure 2I right). This could be due to that immobilization of anti-idiotypic mAb 1G10 by physisorption on the gold electrode potentially led to its partial denaturation resulting in a decreased stability of the capture antibody, which in turn causes signals with poor reproducibility. Overall, the most sensitive layouts were STX assay layout II immobilizing 400 μg/mL anti-idiotypic mAb 1D8 (Figure 2C right), T-2 assay layout I with 100 μg/mL anti-T-2/HT-2 mAb 2A12 as capture antibody (Figure 2F), and AFM1 assay layout I immobilizing 400 μg/mL anti-AFM1 mAb 2D1 (Figure 2I left).

Table 2 summarizes the optimal parameters for the establishment of the competitive electrochemical immunoassay relying on the use of anti-idiotypic mAbs for detection of STX, T-2/HT-2, and AFM1.

**Table 2:** Optimal antibody pair combinations consisting of an anti-idiotypic mAb and an anti-toxin mAb for detection of STX, T-2/HT-2, and AFM1.

**Toxin**	**Assay layout**	**Capture mAb**	**Conc. capture mAb (μg/mL)**	**Detection mAb**	**Conc. detection mAb (ng/mL)**
STX	II	Anti-idiotypic mAb 1D8	400	Anti-STX mAb 5F7-bio	50
T-2/HT-2	I	Anti-T2/HT-2 mAb 2A12	100	Anti-idiotypic mAb 1D6-bio	150
AFM1	I	Anti-AFM1 mAb 2D1	400	Anti-idiotypic mAb 1G10-bio	300

### Analytical Characteristics

**Figure 3:**
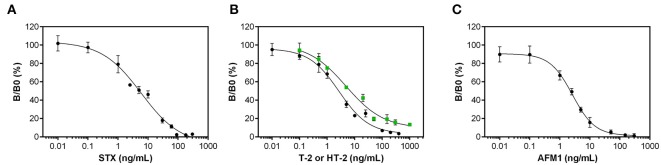
Dose-response curves for the anti-idiotypic antibody-based biochip assays. Measurements with different dilutions of **(A)** STX, **(B)** T-2 (black circles) and HT-2 (green squares), and **(C)** AFM1 were performed in assay buffer. Data presented here were obtained using the optimal antibody pairs described in Table 2. Error bars correspond to the standard deviation obtained from three biochips with two target electrode positions (*n* = 6).

Applying optimal assay parameters determined in the previous chapter, dose-response curves for STX, T-2/HT-2, and AFM1 were generated by analyzing different concentrations of toxins diluted in assay buffer (Figure 3). Correlation of toxin concentration and normalized signal reveals typical sigmoidal shaped curves with negative slope demonstrating that the pairs of anti-toxin mAb and anti-idiotypic mAb function in a competitive way for toxin detection. Analytical parameters obtained from dose-response curves are listed in Table 3.

**Table 3:** Analytical parameters of the anti-idiotypic antibody-based biochip assays for the detection of STX, T-2 and HT-2 as well as AFM1.

**Toxin**	**LOD (ng/mL)**	**IC_**50**_ (ng/mL)**	**Linear working range (ng/mL)**	**CV_**Inter−chip**_ (%)**	**Assay time (min)**
STX	1.0	6.2	0.8–29.7	14.5	16.7
T-2	0.4	2.5	0.4–18.8	7.8	16.7
HT-2	1.0	5.1	1.0–118.6	12.7	16.7
AFM1	0.3	2.4	0.3–8.1	13.1	16.7

#### Dose-Response Curves

The anti-idiotypic antibody-based biochip assay for STX exhibits an LOD in buffer of 1 ng/mL STX with an IC_50_ of 6.2 ng/mL. The linear working range is between 0.8 and 29.7 ng/mL covering two orders of magnitude of concentration above the LOD. Reproducibility of the signal for the individual concentration points between different biochips is expressed as mean coefficient of variation (CV_Inter−chip_) and is 14.5% for the STX assay.

For T-2/HT-2 detection, the dose-response curves reveal a LOD in buffer of 0.4 ng/mL for T-2 and 1 ng/mL for HT-2. The IC_50_ for T-2 and HT-2 are 2.5 ng/mL and 5.1 ng/mL, respectively, indicating that the anti-toxin mAb 2A12 has a higher specificity toward T-2 compared to HT-2. The linear working range covers between two (T-2) and three (HT-2) orders of magnitude of concentration. For both toxins, the CV_Inter−chip_ is below 13% (T-2: 7.8%, HT-2: 12.7%).

To detect AFM1, the anti-idiotypic antibody-based biochip assay has a LOD in buffer of 0.3 ng/mL AFM1 and an IC_50_ of 2.4 ng/mL. The linear working range is between 0.3 and 8.1 ng/mL covering one order of magnitude of concentration. The CV_Inter−Chip_ is 13.1%.

#### Specificity Toward Toxin Group Congeners

**Figure 4:**
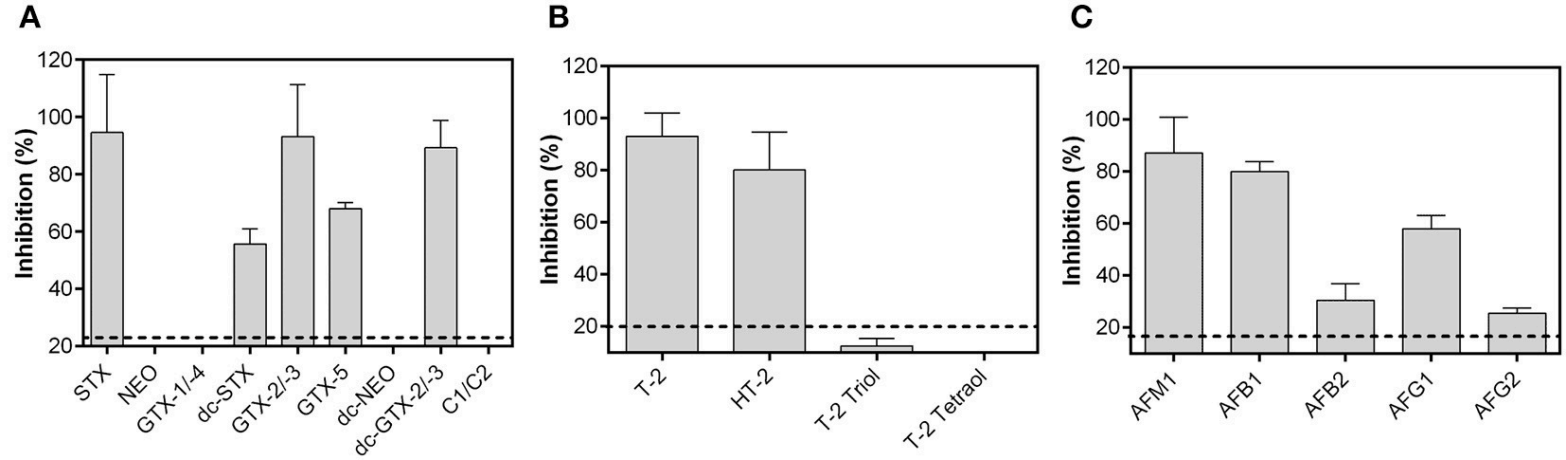
Specificity of the anti-idiotypic antibody-based biochip assays toward toxin group congeners. **(A)** 150 ng/mL PSP toxins, **(B)** 150 ng/mL T-2, and its metabolites and, **(C)** 10 ng/mL aflatoxins were used for analysis. Data presented here were obtained using the optimal antibody pairs described in Table 2. Dashed lines represent the LOD obtained from dose-response curves. Each bar is the mean of the signal from two biochips with two target electrode positions (*n* = 4) depicted as percent inhibition plus its standard deviation.

A broad specificity toward several congeners of a toxin group is required for a biochip assay intended to be used on-site. To assess the potential of each biochip assay to detect a large variety of congeners, respective biochips were exposed to different PSP toxins, T-2 metabolites and aflatoxins. These toxins were used in a concentration close to the lower plateau of the sigmoidal dose-response curves (PSP toxins: 150 ng/mL, T-2 metabolites: 150 ng/mL, aflatoxins: 10 ng/mL). Results are presented in Figure 4 indicating that each biochip assay exhibits a broad specificity toward the respective toxin group.

The STX biochip assay is capable to detect GTX-2/-3, dc-GTX-2/-3, dc-STX, and GTX-5 in addition to STX (Figure 4A). STX, GTX-2/-3, and dc-GTX-2/-3 showed similar levels of signal inhibition, whereas specificity toward dc-STX and GTX-5 is significantly decreased. PSP toxins bearing a hydroxyl group at R1 could not be detected (NEO, dc-NEO, GTX-1/-4, and C1/C2) using the described assay layout.

The T-2/HT-2 biochip assay detects T-2 and HT-2 with a higher sensitivity toward T-2 than toward HT-2 toxin (Figure 4B). Specificity is negligible toward T-2 triol and not-existing toward T-2 tetraol indicating that the acetyl group at C8 and the isovaleric acid group at C15 might be crucial for antibody binding.

The antibody pair for AFM1 detection recognizes all five aflatoxins which are commonly found in food (Figure 4C). The antibodies show the highest sensitivity toward AFM1, followed by AFB1 and AFG1. Specificity toward AFB2 and AFG2, respectively, is clearly weaker than toward AFM1 or AFB1 indicating that the C8-C9 double bond has great influence on antibody affinity.

### Detection of STX, HT-2, and AFM1 in Urine Samples

**Figure 5:**
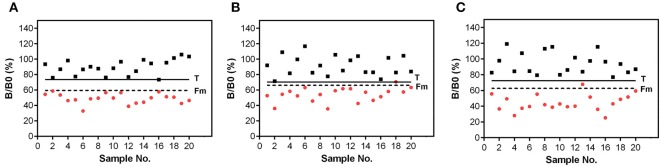
Validation of the biochip assay for detection of **(A)** STX, **(B)** HT-2, and **(C)** AFM1 in urine. Signals were obtained from blank urine (black squares) and urine spiked with target concentration (red circles) of **(A)** 40 ng/mL STX, **(B)** 20 ng/mL HT-2, and **(C)** 8 ng/mL AFM1. Signal in urine was set in relation to the B0 signal in assay buffer. Solid line corresponds to the threshold value T. Dashed line represents the cut-off factor Fm.

To demonstrate the applicability of the assays as rapid screening methods for the detection of STX, HT-2, and AFM1 in urine, the threshold value T and the cut-off factor Fm were assessed for each toxin with an error probability of 5%. Blank urine samples (*n* = 20) and urine samples (*n* = 20) applied at target concentration (STX: 40 ng/mL, HT-2 toxin: 20 ng/mL, AFM1: 8 ng/mL) were analyzed on 40 biochips. Results of the validation are shown in Figure 5.

Validation experiments for the STX biochip assay resulted in a complete separation of 20 blank urine samples and 20 urine samples with a toxin concentration of 40 ng/mL STX (Figure 5A). No false positive as well as no false negative results were observed.

The T-2/HT-2 biochip assay is also applicable for the use in qualitative screenings of urine samples (Figure 5B). Negative urine samples can be distinguished from positive urine samples applied at target concentration of 20 ng/mL HT-2. No false positive results were generated, and one sample (sample 18) is considered as false negative, however, being in line with the error probability of 5%.

The applicability of the biochip assay to detect AFM1 in urine is shown in Figure 5C. A complete separation of blank urine samples and urine samples spiked with 8 ng/mL AFM1 could be achieved. One positive sample (sample 13) is above the cut-off value Fm, but did not exceed the threshold factor T. This sample is considered as non-compliant which is in agreement with the error probability of 5%.

Overall, the cut-off value Fm is markedly below the threshold value T, thus, the three assays are applicable to screen urine samples at or above a target concentration of 40 ng/mL STX, 20 ng/mL HT-2, and 8 ng/mL AFM1.

## Discussion

The developed biochip assays demonstrate that anti-idiotypic antibodies can be applied on an electrochemical biochip for competitive immunoassay-based detection of low molecular weight toxins. The developed assays reveal high sensitivity, rapid assay time, improved assay reproducibility as well as ability for on-site detection provided by the portable detection platform. To our knowledge, an implementation of anti-idiotypic antibody-based assays on electrochemical biochips has not been described so far. The presented data demonstrate that in a competitive immunoassay format the use of potentially harmful toxin-protein conjugates can be avoided and replaced by implementing monoclonal anti-idiotypic antibodies, thus, an environmental-friendly and user-safe assay with a high degree of standardization is generated. Compared to previously reported microarrays for phycotoxin as well as mycotoxin detection using traditional toxin-protein conjugates, the developed assays showed similar or superior assay performance (Supplementary Table 1). Wang et al. (2012) described a microarray using agarose-modified glass slides with fluorescence detection via a bench-top laser scanner for semi-quantitative detection of six mycotoxins in drinking water. LODs for AFM1, AFB1, and T-2 were 0.24, 0.01, and 0.05 ng/mL, respectively. Nevertheless, each step of the assay was performed manually with a total assay time of approximately 240 min. In contrast, our assays implemented on microfluidic biochips overcome these shortages by being automated and more than 14 times faster. More recently, Chen et al. (2018) successfully developed an automated microfluidic microarray to detect four mycotoxins. Detection of T-2 and AFB1 was achieved in 30 min with a prototyping non-portable fluorescence detection platform providing slightly better LODs than the ones reported here. In contrast to our work, toxin-BSA conjugates were immobilized on the microarray, therefore, toxin standards are required for assay production and have to be handled in a safe manner complicating the manufacturing process. Moreover, chemical conjugation to carrier proteins, such as BSA, requires high amounts of toxin standard due to a poor efficiency of the conjugation process (Xiao et al., 1995). Szkola et al. (2013) circumvent this drawback of chemical derivatization by direct immobilization of the native toxin on chemiluminescence microarray chips for detection of three phycotoxins in shellfish within 20 min with LODs in the ng/mL-range. Unlike such microarrays with chemiluminescence read out, electrochemical microarrays do not require luminescence reagents, thus disadvantages, such as the lack of photostability and a low quantum yield, are eliminated. Furthermore, electrochemical microarrays have the advantage that relatively simple, robust and portable read out devices can be used, whereas microarrays using fluorescence or chemiluminescence read out mostly require more sophisticated instrumentation. Uludag et al. (2016) developed an electrochemical readable microchip for detection of AFB1 utilizing an oriented immobilization of anti-toxin antibodies facilitated by protein A bound on the electrode via a self-assembled monolayer. Despite the fact that an oriented immobilization of capture antibodies can lead to an enhanced sensitivity, the here developed assay for AFM1 utilizing a random orientation is up to 10 times more sensitive. Recently, Bratakou et al. (2017) developed a potentiometric STX sensor based on graphene nanosheets electrodes leading to a highly sensitive system with a LOD of 0.3 ng/mL STX. Production of pure graphene sheets is elaborate and quite expensive, whereas the electrical biosensor can be easily and inexpensively manufactured on a large scale (gold electrodes on a low-cost silicon oxide substrate).

A noticeable trend is the simultaneous measurement of multiple toxins in one sample. The configuration of the presented biochip would allow the detection of up to six toxins in duplicates on the same biochip including two Pos.Co. and Neg.Co. positions. Because implementation of anti-idiotypic antibodies in electrochemical immunoassay-based read out platforms has not been described so far, the electrochemical competitive immunoassays for detection of STX, T-2/HT-2, and AFM1 were designed in singleplex formats as a proof-of-concept for the implementation of anti-idiotypic antibodies on electrical biochips.

Moreover, the presented biochip assays in combination with the detection platform exploit key features of electrochemical biochip technology, particularly automatization of assay workflow as well as portability (Farré et al., 2009). Considering the rapid assay time (16.7 min) on a portable platform as well as the high sensitivity of the assays, a promising tool for rapid on-site detection of toxic food contaminants by overcoming the sensitivity limitations of LFAs (Posthuma-Trumpie et al., 2009) was established. A multicolor LFA applying different nanoparticles reported by Xu et al. (2018) enables the parallel detection of AFB1, zearalenone, and T-2 within 20 min with LODs of 0.5, 2, and 30 ng/mL, respectively. There is no specialized read out equipment required; therefore, this LFA is optimally suited for on-site analysis. Nevertheless, sensitivity of the electrochemical biochip method developed in this study showed a slightly improved LOD for AFM1 and a dramatically lower LOD for T-2 applying a similar assay time.

In the past decades, anti-idiotypic antibodies have been most common applied in microplate-based immunoassays. Wang et al. (2013) produced camelid anti-idiotypic heavy chain single domain antibodies and applied these antibodies in a competitive immunoassay format as coating antigen for detection of aflatoxins achieving a sensitive immunoassay setup with an IC_50_ of 0.16 ng/mL for AFB1. However, assay time was approximately 135 min and assay protocol was laborious mainly due to the use of conventional 96-well microplates. The here developed assay utilized automated biochip technology with a minimal hands-on time for the user. Szkola et al. (2014) implemented monoclonal anti-idiotypic antibodies on microarray glass slides to achieve a simultaneous detection of proteotoxins and low molecular weight toxins in the field of biosecurity. The anti-idiotypic antibody was utilized as coating antigen in an indirect assay for detection of STX with an automated chemiluminescence platform. A LOD of 2.3 ng/mL was achieved with an IC_50_ of 10.1 ng/mL and a CV of 28%. The here reported electrochemical detection leads to a 2-fold improvement of the sensitivity (LOD: 1 ng/mL, IC_50_: 6.1 ng/mL). Moreover, the generated current signals were more stable (CV_Inter−chip_: 14.5%) enabling an improved assay reproducibility. In addition to STX, aflatoxins as well as T-2/HT-2 are listed in the Australia group export control list and are regarded as probable candidates for biosecurity screenings (AG, 2018). Thus, applying the developed assays, the robust and fast on-site detection system, pBDi, may also be used as a tool for monitoring these toxins in different samples after deliberate release in the future. Recently, anti-idiotypic antibodies were also utilized in LFAs. Tang et al. (2017) immobilized anti-idiotypic nanobodies on the nitrocellulose membrane of the LFA for detection of AFB1 as well as zearalenone using Eu/Tb (III) nanospheres for time-resolved fluorescence read out. In this study, a LOD of 0.05 and 0.07 ng/mL for AFB1 and zearalenone, respectively, was achieved. In contrast to traditional LFAs, a fluorescence read out requires a portable fluorescence reader for on-site use.

To emphasize the practical application of the developed assays, the biochip assays have been used for qualitative screening of an acute exposure to STX, T-2 and AFB1. It was shown that STX, HT-2 and AFM1 are detected as relevant biomarkers in urine with a low false positive rate (≤5%) in fortified urine samples. The developed STX biochip assay is applicable to detect relevant clinical concentrations of STX in urine. Post-mortem examinations of urine from victims who died after consumption of contaminated shellfish revealed an urinary toxin profile dominated by STX (up to 50%) including up to 1,800 ng/mL STX equivalents (Llewellyn et al., 2002). Moreover, the diagnosis of STX intoxications in patients with suspected PSP was confirmed by detecting STX in urine (Knaack et al., 2016). Recently, Coleman et al. (2018) reported a non-fatal case with an urinary toxin profile dominated by GTX-1/-4 and GTX-2/-3. The urine specimen contained 433 ng/mL GTX-2/-3 (44.8%) and only 15 ng/mL STX (1.7%) demonstrating the necessity for assays exhibiting broad specificity to all PSP toxins. No case reports were published describing an acute exposition to T-2 in combination with the analysis of urinary T-2 metabolites. A first hint assessing HT-2 as biomarker in human biological fluids is provided by a study analyzing human milk samples to estimate mycotoxin exposure to mothers and infants (Rubert et al., 2014). HT-2 was detected in 29% of the samples showing the highest concentration level among the addressed type A trichothecenes (62.5 ng/mL HT-2). The main drawback of HT-2 analysis in urine is the species-dependent elimination route. In animals, elimination occurs within 24 h in feces and urine, whereas *in vivo* studies addressing the elimination route in humans are not available. For the detection of AFM1 in urine, about 1–2% of ingested AFB1 appears as AFM1 in urine (Groopman and Kensler, 1993). Everley et al. (2007) analyzed the urine of a rat administered a dose of AFB1 corresponding to the dose of aflatoxin ingested by human adults during outbreaks in Asia. AFM1 was detected as the major metabolite in urine with a concentration of 48.8 ng/mL indicating that the AFM1 biochip assay presented here can be utilized for detection of relevant concentrations of AFM1 in urine after an aflatoxicosis incident.

These studies clearly illustrate that the presented biochip assays detect the biomarkers STX, HT-2, and AFM1 in relevant concentrations with a low false positive rate in urine samples. Therefore, the presented assays in combination with the on-site detection platform pBDi demonstrate the potential for diagnosing of chronic or acute exposure to low molecular weight toxins by rapid and sensitive detection of relevant biomarkers in urine.

## Conclusion

This paper describes the development of electrochemical biochip assays based on anti-idiotypic antibodies for fast detection of the low molecular weight toxins STX, T-2/HT-2, and AFM1. The biochips were processed in the on-site detection platform pBDi providing a fully automated system with an assay time of 16.7 min. With the optimization of capture and detector antibody concentration, a highly sensitive detection of these toxins with a broad specificity within the toxin group was achieved. The method was proved for the detection of these toxins in urine as biomarkers for toxin exposure. Further work is in progress to implement the singleplex assays for STX, T-2/HT-2, and AFM1 on one biochip allowing multiplex low molecular weight toxin detection as well as development of new reagents enabling improved broad specific detection within the toxin group.

## Author Contributions

KS and CP conceived and designed the experiments for the biochip-based assays. KS performed the experiments for assay implementation on the biochips, analyzed the data and wrote the first draft of the manuscript. CP and EM supervised KS, reviewed and edited the draft of the manuscript. RD conceived and designed the experiments for preparing the monoclonal antibodies. CP, TE, RD, and EM contributed to the writing of the final version of the manuscript. All authors read and approved the final manuscript.

### Conflict of Interest Statement

KS, CP, and TE are current employees of Bruker Daltonik GmbH. Bruker Daltonik GmbH has licensed the electrochemical biochip technology according to EP 1200817 B1 from Fraunhofer Institute for Silicon Technology (Itzehoe, Germany). The remaining authors declare that the research was conducted in the absence of any commercial or financial relationships that could be construed as a potential conflict of interest.

## References

[B1] Adhikari M. Negi B. Kaushik N. Adhikari A. Al-Khedhairy A. A. Kaushik N. K.. (2017). T-2 mycotoxin: toxicological effects and decontamination strategies. Oncotarget 8, 33933–33952. 10.18632/oncotarget.15422 28430618 PMC5464924

[B2] AG (2018). Australia Group Common Control List Handbook Volume II: Biological Weapoons-Related Common Control Lists [Online]. Australia Group. Available online at: https://australiagroup.net/en/documents/Australia-Group-Common-Control-List-Handbook-Volume-II.pdf (Accessed October 24, 2018).

[B3] Anderson D. M. Alpermann T. J. Cembella A. D. Collos Y. Masseret E. Montresor M. (2012). The globally distributed genus Alexandrium: multifaceted roles in marine ecosystems and impacts on human health. Harmful Algae 14, 10–35. 10.1016/j.hal.2011.10.012 22308102 PMC3269821

[B4] Bratakou S. Nikoleli G.-P. Siontorou C. G. Nikolelis D. P. Karapetis S. Tzamtzis N. (2017). Development of an electrochemical biosensor for the rapid detection of saxitoxin based on air stable lipid films with incorporated Anti-STX using graphene electrodes. Electroanalysis 29, 990–997. 10.1002/elan.201600652

[B5] Chanh T. C. Siwak E. B. Hewetson J. F. (1991). Anti-idiotype-based vaccines against biological toxins. Toxicol. Appl. Pharmacol. 108, 183–193. 10.1016/0041-008X(91)90109-R 2017749

[B6] Chen Y. Meng X. Zhu Y. Shen M. Lu Y. Cheng J.. (2018). Rapid detection of four mycotoxins in corn using a microfluidics and microarray-based immunoassay system. Talanta 186, 299–305. 10.1016/j.talanta.2018.04.064 29784365

[B7] Coleman R. M. Ojeda-Torres G. Bragg W. Fearey D. McKinney P. Castrodale L.. (2018). Saxitoxin exposure confirmed by human urine and food analysis. J. Anal. Toxicol. 42, e61–e64. 10.1093/jat/bky031 29800291 PMC6943748

[B8] CRLs (2010). Guidelines for the Validation of Screening Methods for Residues of Veterinary Medicines (Initial Validation and Transfer) [Online]. Community Reference Laboratories Residues (CRLs) 20/1/2010. Available online at: https://ec.europa.eu/food/sites/food/files/safety/docs/cs_vet-med-residues_guideline_validation_screening_en.pdf (Accessed October 08, 2018).

[B9] Dietrich R. Schneider E. Usleber E. Märtlbauer E. (1995). Use of monoclonal antibodies for the analysis of mycotoxins. Nat. Toxins 3, 288–293. 10.1002/nt.2620030423 7582631

[B10] EC (2002). Commission Decision 2002/657/EC of 12 August 2002 Implementing Council Directive 96/23/EC. Official Journal of the European Community L221, 8–36.

[B11] Elsholz B. Worl R. Blohm L. Albers J. Feucht H. Grunwald T.. (2006). Automated detection and quantitation of bacterial RNA by using electrical microarrays. Anal. Chem. 78, 4794–4802. 10.1021/ac0600914 16841897

[B12] Everley R. A. Ciner F. L. Zhan D. Scholl P. F. Groopman J. D. Croley T. R. (2007). Measurement of aflatoxin and aflatoxin metabolites in urine by liquid chromatography-tandem mass spectrometry. J. Anal. Toxicol. 31, 150–156. 10.1093/jat/31.3.150 17579962

[B13] Farré M. Kantiani L. Pérez S. Barceló D. Barceló D. (2009). Sensors and biosensors in support of EU Directives. TrAC Trends Anal. Chem. 28, 170–185. 10.1016/j.trac.2008.09.018

[B14] García C. del Carmen Bravo M. Lagos M. Lagos N. (2004). Paralytic shellfish poisoning: post-mortem analysis of tissue and body fluid samples from human victims in the Patagonia fjords. Toxicon 43, 149–158. 10.1016/j.toxicon.2003.11.018 15019474

[B15] Gessner B. D. Middaugh J. P. Doucette G. J. (1997). Paralytic shellfish poisoning in Kodiak, Alaska. West. J. Med. 167, 351–353. 9392992 PMC1304631

[B16] Granqvist N. Hanning A. Eng L. Tuppurainen J. Viitala T. (2013). Label-enhanced surface plasmon resonance: a new concept for improved performance in optical biosensor analysis. Sensors 13, 15348–15363. 10.3390/s131115348 24217357 PMC3871110

[B17] Groopman J. D. Kensler T. W. (1993). Molecular biomarkers for human chemical carcinogen exposures. Chem. Res. Toxicol. 6, 764–770. 8117914 10.1021/tx00036a004

[B18] Guan D. Li P. Cui Y. Zhang Q. Zhang W. (2011). A competitive immunoassay with a surrogate calibrator curve for aflatoxin M1 in milk. Anal. Chim. Acta 703, 64–69. 10.1016/j.aca.2011.07.011 21843676

[B19] He J. Fan M.-T. Liang Y. Liu X.-J. (2010). Application of anti-idiotype antibody in small molecules immunoassay. Chin. J. Analyt. Chem. 38, 1366–1370. 10.1016/S1872-2040(09)60068-2

[B20] Jerne N. K. (1974). Towards a network theory of the immune system. Ann. Immunol. 125c, 373–389. 4142565

[B21] Knaack J. S. Porter K. A. Jacob J. T. Sullivan K. Forester M. Wang R. Y.. (2016). Case diagnosis and characterization of suspected paralytic shellfish poisoning in Alaska. Harmful Algae 57(Pt B), 45–50. 10.1016/j.hal.2016.03.006 28918891

[B22] Lin Y. Zhou Q. Lin Y. Tang D. Chen G. Tang D. (2015). Simple and sensitive detection of aflatoxin B1 within five minute using a non-conventional competitive immunosensing mode. Biosensors Bioelectron. 74, 680–686. 10.1016/j.bios.2015.07.029 26208172

[B23] Llewellyn L. E. Dodd M. J. Robertson A. Ericson G. de Koning C. Negri A. P. (2002). Post-mortem analysis of samples from a human victim of a fatal poisoning caused by the xanthid crab, *Zosimus aeneus*. Toxicon 40, 1463–1469. 10.1016/S0041-0101(02)00164-2 12368116

[B24] McGrath T. F. Elliott C. T. Fodey T. L. (2012). Biosensors for the analysis of microbiological and chemical contaminants in food. Analyt. Bioanalyt. Chem. 403, 75–92. 10.1007/s00216-011-5685-9 22278073

[B25] McNamee S. E. Elliott C. T. Greer B. Lochhead M. Campbell K. (2014). Development of a planar waveguide microarray for the monitoring and early detection of five harmful algal toxins in water and cultures. Environ. Sci. Technol. 48, 13340–13349. 10.1021/es504172j 25361072

[B26] Nebling E. Grunwald T. Albers J. Schäfer P. Hintsche R. (2004). Electrical detection of viral DNA using ultramicroelectrode arrays. Analyt. Chem. 76, 689–696. 10.1021/ac0348773 14750864

[B27] Nia Y. Rodriguez M. Zeleny R. Herbin S. Auvray F. Fiebig U.. (2016). Organization and ELISA-based results of the first proficiency testing to evaluate the ability of european union laboratories to detect staphylococcal enterotoxin type B (SEB) in buffer and milk. Toxins 8, 268. 10.3390/toxins8090268 27649244 PMC5037494

[B28] Pöhlmann C. Elßner T. (2018). Field-based multiplex detection of biothreat agents, in Enhancing CBRNE Safety & Security: Proceedings of the SICC 2017 Conference, eds Malizia A. D'Arienzo M. (Cham: Springer), 31–39.

[B29] Posthuma-Trumpie G. A. Korf J. van Amerongen A. (2009). Lateral flow (immuno)assay: its strengths, weaknesses, opportunities and threats. A literature survey. Anal. Bioanal. Chem. 393, 569–582. 10.1007/s00216-008-2287-2 18696055

[B30] Rubert J. Leon N. Saez C. Martins C. P. Godula M. Yusa V.. (2014). Evaluation of mycotoxins and their metabolites in human breast milk using liquid chromatography coupled to high resolution mass spectrometry. Anal. Chim. Acta 820, 39–46. 10.1016/j.aca.2014.02.009 24745736

[B31] Shu M. Xu Y. Liu X. Li Y. He Q. Tu Z.. (2016). Anti-idiotypic nanobody-alkaline phosphatase fusion proteins: development of a one-step competitive enzyme immunoassay for fumonisin B1 detection in cereal. Anal. Chim. Acta 924, 53–59. 10.1016/j.aca.2016.03.053 27181644

[B32] Shu M. Xu Y. Wang D. Liu X. Li Y. He Q. (2015). Anti-idiotypic nanobody: a strategy for development of sensitive and green immunoassay for Fumonisin B(1). Talanta 143, 388–393. 10.1016/j.talanta.2015.05.010 26078175

[B33] Szkola A. Campbell K. Elliott C. T. Niessner R. Seidel M. (2013). Automated, high performance, flow-through chemiluminescence microarray for the multiplexed detection of phycotoxins. Anal. Chim. Acta 787, 211–218. 10.1016/j.aca.2013.05.028 23830441

[B34] Szkola A. Linares E. M. Worbs S. Dorner B. G. Dietrich R. Märtlbauer E.. (2014). Rapid and simultaneous detection of ricin, staphylococcal enterotoxin B and saxitoxin by chemiluminescence-based microarray immunoassay. Analyst 139, 5885–5892. 10.1039/c4an00345d 25237676

[B35] Tang X. Li P. Zhang Q. Zhang Z. Zhang W. Jiang J. (2017). Time-resolved fluorescence immunochromatographic assay developed using two idiotypic nanobodies for rapid, quantitative, and simultaneous detection of aflatoxin and zearalenone in maize and its products. Anal. Chem. 89, 11520–11528. 10.1021/acs.analchem.7b02794 28901744

[B36] Turner N. W. Subrahmanyam S. Piletsky S. A. (2009). Analytical methods for determination of mycotoxins: a review. Anal. Chim. Acta 632, 168–180. 10.1016/j.aca.2008.11.010 19110091

[B37] Uludag Y. Esen E. Kokturk G. Ozer H. Muhammad T. Olcer Z.. (2016). Lab-on-a-chip based biosensor for the real-time detection of aflatoxin. Talanta 160, 381–388. 10.1016/j.talanta.2016.07.060 27591628

[B38] Van Egmond H. P. Aune T. Lassus P. Speijers G.J.A. Waldock M. (1993). Paralytic and diarrhoeic shellfish poisons:occurrence in Europe, toxicity, analysis and regulation. J. Nat. Toxins 2, 41–83.

[B39] Vidal J. C. Bonel L. Ezquerra A. Hernandez S. Bertolin J. R. Cubel C.. (2013). Electrochemical affinity biosensors for detection of mycotoxins: a review. Biosens Bioelectron 49, 146–158. 10.1016/j.bios.2013.05.008 23743326

[B40] Wang Y. Li P. Majkova Z. Bever C.R.S. Kim H. J. Zhang Q.. (2013). Isolation of alpaca anti-idiotypic heavy chain single domain antibody for the aflatoxin immunoassay. Anal. Chem. 85, 8298–8303. 10.1021/ac4015885 23965250 PMC3787825

[B41] Wang Y. Liu N. Ning B. Liu M. Lv Z. Sun Z.. (2012). Simultaneous and rapid detection of six different mycotoxins using an immunochip. Biosensors Bioelectron. 34, 44–50. 10.1016/j.bios.2011.12.057 22341860

[B42] Williams J. H. Phillips T. D. Jolly P. E. Stiles J. K. Jolly C. M. Aggarwal D. (2004). Human aflatoxicosis in developing countries: a review of toxicology, exposure, potential health consequences, and interventions. Am. J. Clin. Nutr. 80, 1106–1122. 10.1093/ajcn/80.5.1106 15531656

[B43] Worbs S. Skiba M. Bender J. Zeleny R. Schimmel H. Luginbuhl W.. (2015). An international proficiency test to detect, identify and quantify ricin in complex matrices. Toxins (Basel) 7, 4987–5010. 10.3390/toxins7124859 26703726 PMC4690109

[B44] Wu Q. Dohnal V. Huang L. Kuca K. Yuan Z. (2010). Metabolic pathways of trichothecenes. Drug Metab. Rev. 42, 250–267. 10.1080/03602530903125807 19678805

[B45] Xiao H. Clarke J. R. Marquardt R. R. Frohlich A. A. (1995). Improved methods for conjugating selected mycotoxins to carrier proteins and dextran for immunoassays. J. Agric. Food Chem. 43, 2092–2097. 10.1021/jf00056a025

[B46] Xu L. Zhang Z. Zhang Q. Zhang W. Yu L. Wang D.. (2018). An on-site simultaneous semi-quantification of aflatoxin B1, zearalenone, and T-2 toxin in maize- and cereal-based feed via multicolor immunochromatographic assay. Toxins 10:87. 10.3390/toxins10020087 29462999 PMC5848188

