# Supplementary material for: Electrochemical Biochip Assays Based on Anti-idiotypic Antibodies for Rapid and Automated On-Site Detection of Low Molecular Weight Toxins
Source: Front Chem. 2019 Feb 1;7:31. doi: 10.3389/fchem.2019.00031 (PMC6367258; doi:10.3389/fchem.2019.00031)
Supplement: Supplementary file 1 [file Table_1.DOCX]

Supplementary Material

Electrochemical biochip assays based on anti-idiotypic antibodies for rapid and automated on-site detection of low molecular weight toxins

Katharina Schulz, Christopher Pöhlmann, Richard Dietrich, Erwin Märtlbauer*, Thomas Elßner

***Correspondence:**Erwin Märtlbauer
[e.maertlbauer@mh.vetmed.uni-muenchen.de](mailto:e.maertlbauer@mh.vetmed.uni-muenchen.de)

Supplementary Table 1: Comparison of the developed anti-idiotypic antibody based biochip with biosensors using traditional toxin-protein conjugates to detect phycotoxins as well as mycotoxins. Automatization and portability are solely based on what is reported by the authors, although it may be possible that all the methods can be engineered in order to have these characteristics.

| **Toxin** | **Biosensor surface** | **Detection principle** | **LOD in buffer (ng/mL)** | **Assay time (min)** | **Integration in a detection platform** | | **Application** | **Reference** |
| --- | --- | --- | --- | --- | --- | --- | --- | --- |
|  |  |  |  |  | **Fully automated** | **Portable** |  |  |
| STX | Singleplex microarray on gold electrode with anti-idiotypic mAb as capture | Electrochemical | 1 | 16.7 | Yes | Yes | Urine | Our study |
| STX  DA  OA | Multiplex microarray on glass slide with toxin as capture | Chemilumi-nescence | 1  0.5  0.3 | 20 | Yes | Yes | Food | Szkola et al. (2013) |
| STX | Singleplex graphene nanosheet electrode with anti-STX pAb as capture incorporated into lipids | Electrochemical | 0.3 | 5-20 | Yes | No | Water,  food | Bratakou et al. (2017) |
| T-2  HT-2 | Singleplex microarray on gold electrode with anti-T-2/HT-2 mAb as capture | Electrochemical | 0.4  1.5 | 16.7 | Yes | Yes | Urine | Our study |
| AFM1 | Singleplex microarray on gold electrode with anti-AFM1 mAb as capture | Electrochemical | 0.3 | 16.7 | Yes | Yes | Urine | Our study |
| T-2  AFB1  OTA  ZEN | Multiplex microarray on glass slide with BSA-toxin conjugate as capture | Fluorescence | 0.12  0.03  1.24  0.58 | 30 | Yes | No | Water,  feed | Chen et al. (2018) |
| AFB1 | Singleplex microarray on gold electrode with Protein A coupled to SAM | Electrochemical | 2 | 25 | Yes | Yes | Food | Uludag et al. (2016) |
| AFM1  AFB1  T-2  DON  OTA  ZEN | Multiplex microarray on agarose-modified glass slide with BSA-toxin conjugate as capture | Fluorescence | 0.24  0.01  0.05  15.45  15.39  0.01 | 240 | No | No | Water | Wang et al. (2012) |
| Abbreviations: DA = Domoic acid; OA = Okadaic acid; OTA = Ochratoxin; ZEN = Zearalenone; DON = Deoxynivalenol; SAM = Self-assembled monolayer; BSA = Bovine serum albumin; mAb = Monoclonal antibody; pAb = Polyclonal antibody | | | | | | | | |

References

Bratakou, S., Nikoleli, G.-P., Siontorou, C.G., Nikolelis, D.P., Karapetis, S., and Tzamtzis, N. (2017). Development of an Electrochemical Biosensor for the Rapid Detection of Saxitoxin Based on Air Stable Lipid Films with Incorporated Anti-STX Using Graphene Electrodes. *Electroanalysis* 29(4)**,** 990-997. doi: 10.1002/elan.201600652.

Chen, Y., Meng, X., Zhu, Y., Shen, M., Lu, Y., Cheng, J., et al. (2018). Rapid detection of four mycotoxins in corn using a microfluidics and microarray-based immunoassay system. *Talanta* 186**,** 299-305. doi: 10.1016/j.talanta.2018.04.064.

Szkola, A., Campbell, K., Elliott, C.T., Niessner, R., and Seidel, M. (2013). Automated, high performance, flow-through chemiluminescence microarray for the multiplexed detection of phycotoxins. *Anal. Chim. Acta* 787(1)**,** 211-218. doi: 10.1016/j.aca.2013.05.028.

Uludag, Y., Esen, E., Kokturk, G., Ozer, H., Muhammad, T., Olcer, Z., et al. (2016). Lab-on-a-chip based biosensor for the real-time detection of aflatoxin. *Talanta* 160**,** 381-388. doi: 10.1016/j.talanta.2016.07.060.

Wang, Y., Liu, N., Ning, B., Liu, M., Lv, Z., Sun, Z., et al. (2012). Simultaneous and rapid detection of six different mycotoxins using an immunochip. *Biosensors and Bioelectronics* 34(1)**,** 44-50. doi: 10.1016/j.bios.2011.12.057.
